# Dysregulated metabolic homeostasis as a unifying death mechanism underlying the diverse clinical manifestations of COVID-19: insights from a retrospective analysis of sequential blood variables

**DOI:** 10.3389/fmed.2026.1773937

**Published:** 2026-03-12

**Authors:** Zvia Agur, Yuri Kogan, Anat Ben Yaacov, Edward Itelman, Gad Segal

**Affiliations:** 1 Institute for Medical Biomathematics, Bene Ataroth, Israel; 2 Department of Internal Medicine, Chaim Sheba Medical Center, Faculty of Medicine, Tel Aviv University, Tel Aviv, Israel

**Keywords:** ARDS, glycolysis, hemostasis, hypercoagulability, lactic acidosis, metabolic homeostasis, metabolic reprogramming, mitochondriopathy

## Abstract

**Background:**

COVID-19 presents diverse clinical manifestations associated with increased mortality, yet a unifying death mechanism remains elusive; here, we suggest such a mechanism that implies a simple way to lower deaths. This work differs from previous studies that use machine learning to identify mortality predictors.

**Methods:**

Viewing clinical deterioration to a severe stage as a distinct “junction” in disease progression, we collected 173 medical records of COVID-19 patients who deteriorated and divided them into two groups: those who died (nonsurvivors) and those who recovered after deterioration (survivors). We aligned patients’ medical records by clinical deterioration time and statistically compared the two groups using standard blood variables.

**Results:**

Significant differences between the groups emerged only in the first week after clinical deterioration: nonsurvivors showed a rapid, simultaneous rise in lactate dehydrogenase (*p* ≤ 0.0001) and D-dimer (*p* ≤ 0.0001), followed by a decrease in platelet counts in the second week (*p* ≤ 0.0001). Other variables remained consistent throughout hospitalization. Older patients showed similar but less significant response patterns. Based on these clinical results, we hypothesized that the mechanism of death in COVID-19 involves an abrupt glycolytic surge during deterioration, driven by concurrent hypoxemia and virus-induced mitochondriopathy, resulting in significant disruption of metabolic homeostasis, which leads to imbalanced hemostasis and death.

**Conclusion:**

Our findings highlight the importance of timing in COVID-19 treatment. Using an available machine learning algorithm to predict imminent deterioration enables prompt, short-term intervention with prophylactic mechanical ventilation and optimal antiglycolytic therapy. Implementing this approach requires further experimental and clinical validation. Identifying metabolism-related genetic or epigenetic anomalies in nonsurvivors will support our hypothesis and aid in classifying the high-risk patients.

## 1 Introduction

Numerous pathological mechanisms are brought up as contributing to the death of patients with COVID-19. Among the most frequent mortality-associated pathologies are acute respiratory distress syndrome (ARDS), Diffuse Alveolar Damage, Microthrombosis, Cytokine Storm, Superimposed Bacterial and Fungal Infections, Cardiovascular Complications, and Multiple Organ Failure (MOF) (1–6). These pathologies are characterized by a harsh, dysregulated systemic inflammatory response that activates immune and endothelial cells, causing widespread tissue and vascular damage, microvascular thrombosis, and impaired organ function (7, 8). One may ask whether the various clinical syndromes observed in COVID-19 are distinct events or, instead, stem from a common underlying cause. If the latter is true, identifying that shared cause could be a key step toward an effective cure for this complex inflammatory disease.

Several glycolytic biomarkers, especially lactate dehydrogenase (LDH), have demonstrated significant predictive value for identifying COVID-19 patients at higher risk of death, particularly those admitted to intensive care units (ICU). Recent studies indicate that LDH alone, or combined with IL-6 and lymphocyte count, is the most effective laboratory indicator of COVID-19 mortality risk (9, 10). Additionally, high plasma levels of the glycolysis byproduct methylglyoxal (MG) at ICU admission with ARDS were shown to predict death in COVID-19 patients. It has been suggested that an increase in MG results from heightened glycolysis in individuals with COVID-19 (11). Another glycolytic product, lactate, also serves as a potential biomarker in COVID-19, with significantly elevated blood levels observed in nonsurvivors compared to survivors (12). Indeed, increased glycolysis is associated with COVID-19 severity and mortality, as it plays a crucial role in excessive innate immune activation and viral replication within infected cells. However, the direct causal relationship between glycolysis and death in COVID-19 patients remains unclear. Understanding this relationship could improve the efficacy of anti-glycolytic therapies for this disease, which currently show mixed results in clinical trials. It may also lead to the development of new treatment options for COVID-19 (13–16).

The present study seeks to investigate the underlying death mechanism in COVID-19 by analyzing the dynamics of standard blood variables in COVID-19 patients who progressed from mild or moderate to severe illness. By limiting the sample to patients who clinically deteriorated to severe disease, we could identify differences among patients at the same disease stage. Our findings highlight significant differences in metabolic and hemostatic features between survivors and nonsurvivors. We propose a mechanism-based framework to explain these features and call to examine a simple treatment approach to reduce mortality rates.

## 2 Materials and methods

### 2.1 Clinical data

We analyzed medical records of hospitalized patients at Sheba Medical Center. The study was approved by the Sheba Medical Center Institutional Review Board–Helsinki Committee (#7953-20-SMC) in accordance with the Declaration of Helsinki. All data were anonymized and handled in accordance with ethical and regulatory standards, with informed consent waived due to the study’s retrospective design.

We included all patients hospitalized between March 2020 and August 2021 who met predefined criteria (as detailed in the following subsection), forming a cohort of 173 COVID-19 patients infected with the dominant variants at the time. From anonymized Electronic Medical Records, we extracted longitudinal data on seven blood variables—lymphocyte, neutrophil, monocyte, and platelet counts, and CRP, LDH, and D-dimer levels. In addition, daily clinical status assessments were extracted for each patient. These assessments evaluated the disease severity on the standard 4-grade scale: mild, moderate, severe, or critical.

### 2.2 Inclusion criteria

The study included 173 patients initially classified as mild or moderate at admission who progressed to severe or critical status within 3 weeks. Only patients with at least one blood test between admission and 21 days after clinical deterioration were analyzed. For each patient, dynamic features were calculated over defined time intervals, provided there were enough tests within each interval.

### 2.3 Longitudinal comparison

We defined the time point of clinical deterioration as the first time a patient’s status became severe or critical and remained at that level for at least 72 h. All the patients were aligned on a timeline relative to their clinical deterioration event (*t* = 0). For each variable, measurements from all patients within 7 and 21 days before and after clinical deterioration, respectively, were grouped into two categories: survivors and nonsurvivors. It is important to note that many patients deteriorated within 14 days after admission, which is why it was necessary to evaluate patients’ dynamics only during the week before deterioration. Moving local regression curves were estimated for each group using locally estimated scatterplot smoothing (loess) in R Stats v4.2.2, as shown in Figure 2A (blue for survivors; red for nonsurvivors). Standard errors are displayed as dashed lines, with 25–75% (dark shading) and 2.5–97.5% (light shading) confidence intervals estimated through bootstrapping, and dotted lines indicating bootstrap medians. To analyze age-related dynamics, patients were divided into two subgroups: those younger than 70 and those aged 70 or older at admission.

### 2.4 Computation and comparison of dynamic features

For each variable, we defined 14 dynamic features that evaluate the average values and changes over specific time intervals for each patient. The features included averages over the following time intervals, measured in days: (-7, 0), (-3, 0), (0, 3), (0, 7), (-3, 3), (-7, 7), (-14, -7), and (7,14); maximal change (defined as the signed difference with the maximal absolute value from the measurement at *t* = 0) over the time intervals (0, 3), (0, 7), and (7, 14); change in average (defined as the difference between the average over the given interval and the average over the pre-deterioration interval of the same length) over the time intervals (0, 3), (0, 7), and (7, 14). Note that results from more than 7 days before deterioration are not presented in the figures and tables, as most patients deteriorated within 2 weeks of admission. For each feature, we computed the Mann-Whitney U statistic and its *p*-value, and the ROC AUC (Receiver Operating Characteristic Area Under the Curve) for discrimination between survivors and nonsurvivors. The ggplot2 package in R generated the violin plots, boxplots, averages, S.D. lines, and point scatters. To determine the significance threshold, we employed the Bonferroni correction: ρ≤ a/m, where ρ is the desired *p*-value of the null hypothesis, a is the desired significance level, and *m* is the number of tests performed. The significance level was set at a = 0.01.

## 3 Results

### 3.1 The dynamics of blood variables at clinical deterioration differ between survivors and nonsurvivors

**FIGURE 1:**
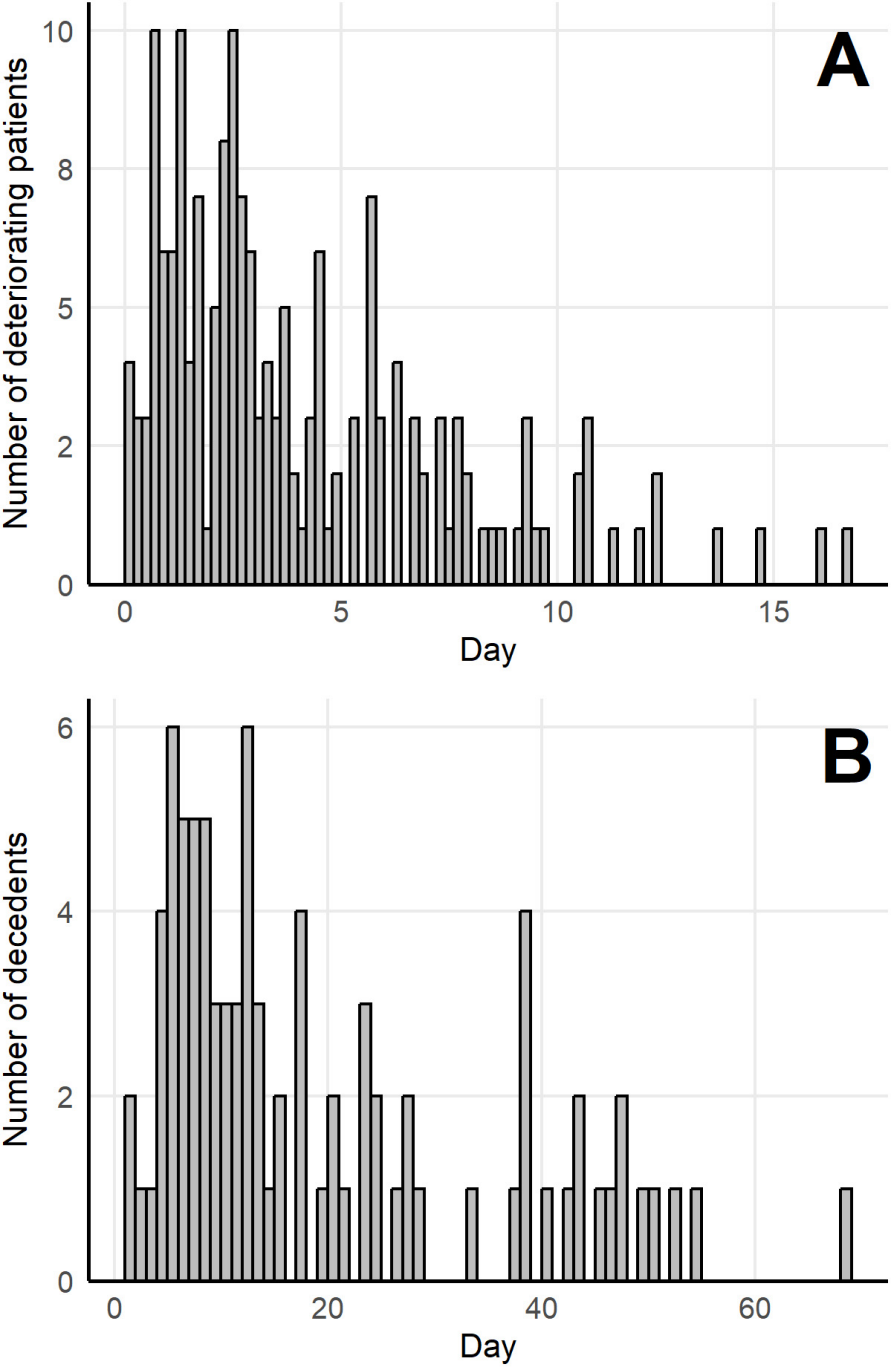
Distribution of deterioration and death times in a cohort of 173 deteriorating patients with COVID-19. **(A)** The number of deteriorating patients per day after admission (taken as day 0). **(B)** The number of patients dying per day after deterioration (taken as day 0).

Respiratory dysfunction and hypoxia are the most significant signs of clinical deterioration in COVID-19, identified by SpO_2_ < 94%, a respiratory rate of 30 breaths per minute or more, or lung infiltrates covering more than 50% on a routine chest X-ray. In our patient cohort, 173 individuals deteriorated from mild or moderate state to severe or critical state, with a median time to deterioration of 2.89 days after hospital admission (Figure 1A). Eighty-seven of these patients survived at least ten weeks after clinical deterioration (called survivors); 86 patients died within ten weeks after clinical deterioration, with a median time to death of 12.93 days (called nonsurvivors; Figure 1B). As shown in Figure 1B, the highest weekly death toll occurred during the first 2 weeks after clinical deterioration.

**FIGURE 2:**
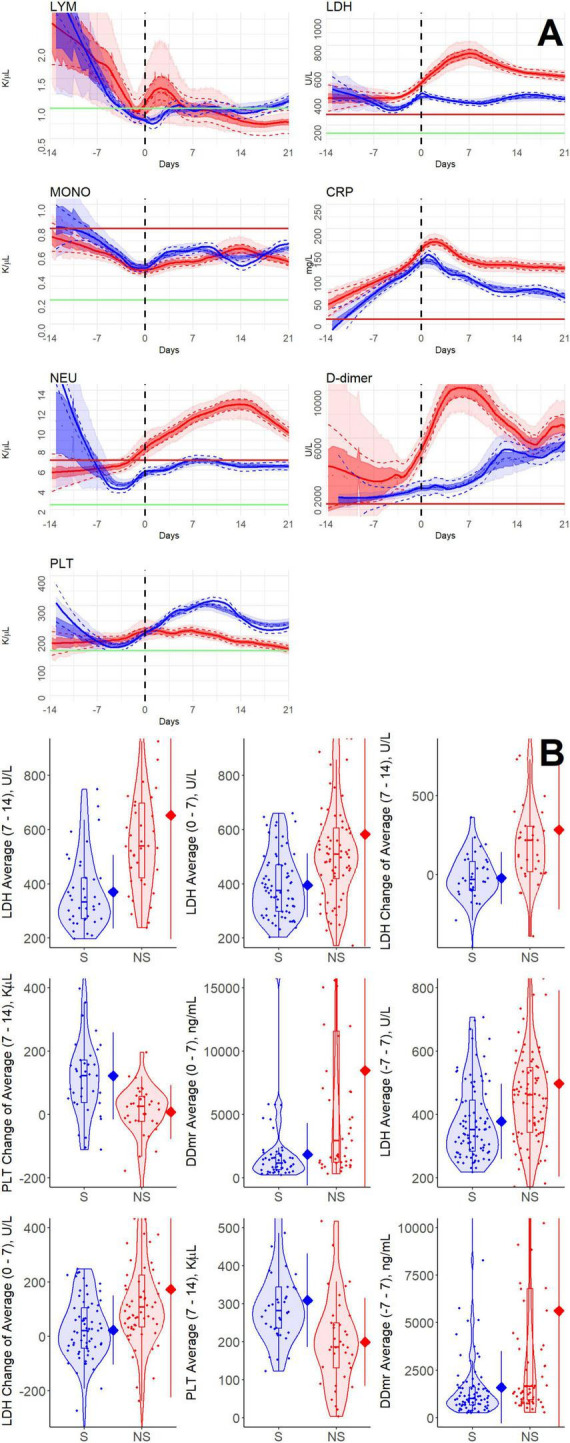
Longitudinal measurements of blood variables in a cohort of 173 patients with COVID-19 who are deteriorating. **(A)** Moving local regression of blood variables within the studied cohort (solid curves) and their standard error (dashed curves), estimated by the loess algorithm. The vertical dashed black line indicates the time of deterioration, set to *t* = 0. For each group, the central dotted curves show the median, and shaded areas represent the 25–75% and 2.5–97.5% confidence intervals around the median, estimated via bootstrap (darker and lighter shading, respectively). Green and red horizontal lines denote the lower and upper limits of normal ranges for the laboratory values, respectively. Blue: survivor group; red: nonsurvivor group. Blood variables are denoted as follows: LYM, lymphocytes; MONO, monocytes; NEU, neutrophils; PLT, platelets; LDH, lactate dehydrogenase; CRP, C-reactive protein; D-dimer is measured in fibrinogen equivalent units (FEU). **(B)** Distributions of blood variable features for survivors (blue) and nonsurvivors (red). The width of each violin plot reflects the smoothed density of data points in each region. Box plots within each violin show the first and third quartiles, with a central horizontal line indicating the median. Rhombi on the right vertical bar represent mean values of the feature, and line lengths indicate standard deviation.

Figure 2A displays the smoothed averages and confidence intervals of longitudinal measurements for the selected blood variables in the patient groups of survivors and nonsurvivors, aligned relative to the time of deterioration (*t* = 0). The results in Figure 2A indicate that before clinical deterioration, lymphocyte, monocyte, and neutrophil counts steadily declined with little difference between the survivors’ and nonsurvivors’ groups. Following clinical deterioration, the average counts of lymphocytes and monocytes remained relatively low in both survivors and nonsurvivors, whereas neutrophils increased in the nonsurvivors’ group. As shown below, these blood counts do not demonstrate any statistically significant differences between the survivor and nonsurvivor groups during the disease progression. Platelet counts of survivors and nonsurvivors showed different patterns. The survivor group displayed an oscillating pattern within the normal range. In contrast, the nonsurvivors group’s platelet counts generally declined toward thrombocytopenia, with a slight increase around the time of clinical deterioration (17).

The biochemical variables LDH, D-dimer, and CRP were above their normal ranges in all patients, largely overlapping between the survivor and nonsurvivor groups before clinical deterioration and increased further at the time of clinical deterioration. A notable difference between the survivor and nonsurvivor groups emerged in the LDH and D-dimer trends over the 2 weeks following clinical deterioration. The nonsurvivors group experienced a sharp rise, while the survivor group showed a roughly steady pattern. This contrasts with the CRP profile, which showed similar trends in both groups. However, it is important to note that CRP values consistently remained somewhat higher in the nonsurvivors group.

### 3.2 Following clinical deterioration, survivors and nonsurvivors manifested significantly different dynamic features of LDH, platelets, and D-dimer

To examine the significance and discriminative power of the observed differences between the two patient groups, we defined a set of metrics that quantify diverse, dynamic features of the longitudinal measurements. We statistically evaluated differences between the survivor and nonsurvivor groups for all examined variables at 3, 7, or 14 days, pre- or post-deterioration (Figure 2B and Tables 1, 2).

**TABLE 1:** Significance of the *U*-test comparing the features of the survivors’ and nonsurvivors’ blood variables in the deteriorating patient cohort.

Value (days)	LYM	NEU	MONO	PLT	CRP	LDH	D-dimer
Average (-7–0)	*	–	–	–	–	*	–
Average (-3–0)	**	–	*	–	–	*	*
Average (0–3)	–	*	*	–	–	**	***
Average (0–7)	**	**	**	**	**	****	****
Average (7–14)	**	***	–	****	**	****	***
Average (-3–3)	*	*	*	–	–	***	***
Average (-7–7)	**	*	*	*	*	****	****
Max change (0–3)	–	–	–	*	–	*	–
Max change (0–7)	–	–	–	***	–	**	*
Max change (7–14)	–	**	–	***	–	***	**
Change of average (0–3)	–	–	*	**	–	**	–
Change of average (0–7)	–	–	–	***	*	****	*
Change of average (7–14)	*	**	–	****	–	****	**

Statistical significance of dynamic blood features in distinguishing between surviving and non-surviving patients with COVID-19 who are deteriorating. The significance levels of differences between survivor and nonsurvivor groups in the dynamic features of blood variables during the 7 days before and the 14 days after deterioration are shown. The statistically significant features, as determined by the U-test, applying the Bonferroni correction, are marked by rectangles;

*p ≤ 0.05,

**p ≤ 0.01,

***p ≤ 0.001,

****p ≤ 0.0001, for notations of blood variable see Figure 2.

**TABLE 2:** ROC AUC and *p* values of the U–test for the most significant blood variable features differentiating between survivors and non-survivors in the deteriorating patient cohort.

Feature (days)	ROC AUC	*p*	N_*S*_	N_*D*_
LDH average (7–14)	0.802	2.462 × 10^–06^	38	38
LDH average (0–7)	0.712	1.359 × 10^–05^	71	70
LDH change in average (7–14)	0.785	2.265 × 10^–05^	34	36
PLT change in average (7–14)	0.776	5.135 × 10^–05^	36	37
D-dimer average (0–7)	0.737	5.365 × 10^–05^	47	51
LDH average (-7–7)	0.678	5.458 × 10^–05^	85	87
LDH change in average (0–7)	0.703	6.084 × 10^–05^	65	66
PLT average (7–14)	0.76	6.537 × 10^–05^	37	39
D-dimer average (-7–7)	0.687	7.945 × 10^–05^	70	80
PLT Max change (0–7)	0.681	0.0002	70	70
D-dimer average (0–3)	0.747	0.0003	32	40
PLT change in average (0–7)	0.679	0.0004	66	65
LDH average (-3–3)	0.659	0.0004	82	83
LDH Max change (7–14)	0.732	0.0005	38	38
D-dimer average (7–14)	0.75	0.0005	28	33
D-dimer average (-3–3)	0.671	0.0007	59	75
PLT Max change (7–14)	0.726	0.0007	37	39
NEU average (7–14)	0.722	0.0009	37	39

Significance and differentiation capacity of the most discriminative blood features between surviving/non-surviving deteriorating COVID-19 patients. The features are presented in descending order of significance (*p*-values) for the difference between survivors and nonsurvivors as determined by ROC AUC and U-test *p*-values. The eighteen most significant features are shown. The horizontal thick line marks the partition between the significant *p*-values (above the line) and the non-significant values (below the line), as determined by the U-test, applying the Bonferroni correction (see Methods); N_*S*_ and N_*D*_ denote numbers of survivors and nonsurvivors, respectively, for whom the feature value could be computed. Other details appear in the legends of Table 1.

In Figure 2B, we show violin plots of blood variable features for individual survivors and nonsurvivors (see Methods). The width of each violin plot indicates the distribution density, while the dots represent the actual data points. The box plots within the violins span from the first to the third quartiles, with a horizontal line in the middle indicating the median. A rhombus on the right-side vertical bar of each violin plot represents the average value of the group, and the bar indicates the standard deviation (S.D.). The displayed features have, in general, non-normal distributions, as shown by the shape of the violin plots. We used the Mann–Whitney U-test (which is appropriate for non-normal distributions) to compare the dynamic features of survivors and nonsurvivors (see Methods). The U-test results indicate that, prior to clinical deterioration, dynamic blood features did not differ between survivors and nonsurvivors. However, several LDH and D-dimer features during the first 2 weeks after deterioration, along with average platelet counts in the second week after deterioration, significantly differed between the survivor and nonsurvivor groups (Table 1).

Table 2 shows the ROC AUC values and the U-test *p*-values for the most important features, listed in order of decreasing significance. The significance threshold, set at 10^−4^, is highlighted with a thick horizontal bar (see Methods). LDH levels in the 2 weeks following clinical deterioration show the most pronounced difference between the nonsurvivor and survivor groups. The average D-dimer measurements during the first week after clinical deterioration also display highly significant differences between the two groups. The difference in average platelet counts during the second week post-deterioration compared to the previous week is also significant. Interestingly, although there is a peak in neutrophil counts (neutrophilia) in the nonsurvivor group during the second week after clinical deterioration, and not in the survivor group, this does not seem to be a significant discriminator at the chosen threshold.

### 3.3 Different age groups show similar dynamic patterns

To assess the generalizability of our results across patient ages, we divided our cohort into two subcohorts: patients younger than 70 years (*N* = 68) and patients aged 70 or older (*N* = 105). Results from both subcohorts showed similarity to the dynamic patterns observed in the whole cohort (not shown). In the two sub-cohorts, the differences between the survivors and nonsurvivor group averages were mainly in the levels of LDH, D-dimer, and platelets in the first 2 weeks after clinical deterioration, even though the differences in LDH and platelets between the survivors and the non-survivor groups were significant only in the younger sub-cohort. This difference may be due to reduced stabilization of the transcription factor that acts as a master regulator of cellular adaptation to low oxygen—the Hypoxia-inducible factor-1α (HIF-1α), (see below), and, as a result, reduced glycolytic response in older people, which could be contributing to age-related conditions, such as diabetes (18). Additionally, note that a relatively small cohort size limits the statistical significance of potential findings within age subgroups.

### 3.4 A theoretical model for deterioration-associated death in COVID-19

#### 3.4.1 Our associative findings serve as a foundation for mechanistic model hypotheses

We briefly summarize the study’s associative findings and detail their role in forming a new hypothesis about the mechanism of death in COVID-19. We compared longitudinal patterns in blood variables between survivors and nonsurvivors in a cohort of patients who experienced clinical deterioration. These statistical analyses revealed no significant differences during the mild and moderate stages of the disease. In contrast, significant differences appeared during clinical deterioration and the following 2 weeks, when most patient deaths occurred. The blood variables that increased significantly and sharply in the nonsurvivors group were LDH and D-dimer. Additionally, platelet counts appeared to fluctuate but remained within their normal ranges in the survivor group, whereas in nonsurvivors, platelet counts tended to be thrombocytopenic, with a transient increase at deterioration (Figure 2).

Previous studies have indicated direct or indirect associations between the aforementioned blood variables, disease progression, hypoxia, and metabolic changes. Clinical deterioration is characterized by hypoxemia, tachypnea, and often new or worsening dyspnea. Tachypnea and dyspnea are non-specific markers of respiratory or metabolic stress, for which hypoxia/hypoxemia are important differential diagnoses (19). Elevated serum LDH levels in COVID-19 patients are associated with increased glycolysis (hyperglycolysis) in infected cells and tissues, as well as systemic metabolic reprogramming toward anaerobic metabolism (15) (but see further discussion below). In COVID-19, enhanced glycolysis in platelets is known to contribute to their activation, procoagulant shift, and eventual depletion, often leading to thrombocytopenia (20). A procoagulant shift promotes excessive thrombin and fibrin formation, leading to increased cross-linked fibrin clots. Plasmin then degrades these clots, releasing D-dimer as a byproduct, which raises measurable plasma levels (21). D-dimer is primarily a marker of coagulation activation and fibrinolysis (22, 23). In COVID-19, hypoxia exacerbates hypercoagulability and venous thromboembolism risk through intertwined mechanisms involving endothelial damage, inflammation, and impaired fibrinolysis (24).

#### 3.4.2 Integrating the associative findings with published evidence: a new hypothetical death mechanism

**FIGURE 3:**
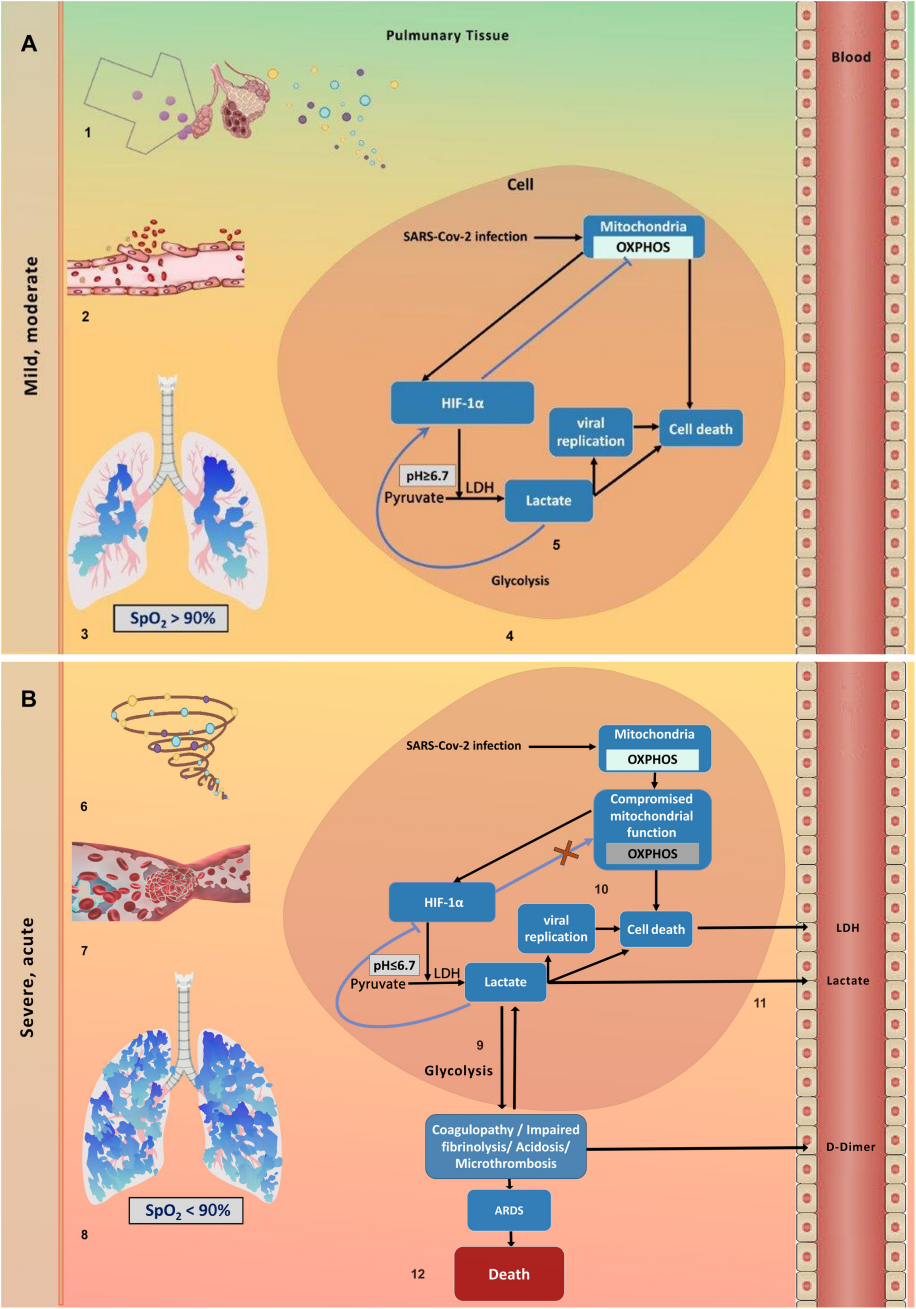
A conceptual model for deterioration-associated death in COVID-19. **(A)** Mild and moderate disease stage. 1. The SARS-CoV-2 virus triggers antiviral immune responses in lung epithelial cells, upregulating the release of proinflammatory cytokines into the extracellular space (45). 2. This inflammatory response damages adjacent microvascular endothelial cells (45). 3. It further causes uneven capillary perfusion and hypoxic alveolocapillary units (46, 47). 4. At the cellular level, hypoxia triggers metabolic reprogramming from oxidative phosphorylation (OXPHOS) to anaerobic glycolysis, downregulating OXPHOS-associated genes and upregulating glycolysis-associated genes (48). 5. Lactate is converted from pyruvate by LDH and accumulates at low concentrations (pH > ∼6.7), stabilizing the key transcription factor which controls metabolic homeostasis, Hypoxia Inducible Factor 1 α (HIF-1α), by a positive feedback effect, which boosts glycolysis and speeds up viral replication (29, 49). **(B)** Deterioration and severe and critical disease stages. 6. Accelerated SARS-CoV-2 replication causes an uncontrolled inflammatory response, with hypercytokinemia (50). 7. This hyperinflammation contributes to coagulopathy and microvascular thrombosis in the pulmonary circulation. Inadequate oxygenation and increased blood viscosity further promote microvascular thrombosis and hypoxia (49). 8. At a crucial moment, clinically defined as *deterioration*, the oxygen level in the lungs falls below the hypoxemia threshold (SpO_2_∼90%), and the extracellular lung milieu becomes severely hypoxemic. Hypoxemia considerably increases cell mortality and promotes a shift to glycolysis in most of the remaining uninfected living cells in the lungs (51). 9. The massive glycolysis occurring simultaneously in the lung tissue produces large amounts of extracellular lactate that leak into cells, lowering intracellular pH below 6.7. This triggers lactate-mediated negative feedback on glycolysis, destabilizing HIF-1α and shifting cellular metabolism back toward OXPHOS (52, 53). 10. However, the replicating SARS-CoV-2 viruses cause rapid and widespread mitochondriopathy, leading to apoptosis in alveolar epithelial and airway cells, as well as in pulmonary artery smooth muscle and endothelial cells (54, 55). 11. We postulate that due to the substantial lung mitochondriopathy, OXPHOS is suppressed, trapping cells in excessive glycolysis, which further increases lactic acidosis and raises LDH and D-dimer levels; these are secreted into the blood from damaged cells. 12. We further postulate that at this deterioration stage, excessive glycolysis decreases respiratory system compliance, leading to pulmonary embolism and microcirculatory changes, which worsen the clinical course toward acute respiratory distress syndrome (ARDS) and lower patient survival rates (34, 56–58).

Gleaned from the above evidence, we hypothesized (i) that death from COVID-19 results from acute hypoxia in lung tissue during disease deterioration, causing extensive metabolic reprogramming from oxidative phosphorylation (OXPHOS) to glycolysis, (ii) and that SARS-CoV-2–induced mitochondrial dysfunction in nonsurvivors hinders the shift back to OXPHOS, forcing cells to rely on glycolysis and leading to marked lactate accumulation. Integrating our hypotheses with further evidence from the literature, we assume the following sequence of events leading to deterioration-associated death in COVID-19. A more detailed description of our conceptual model, including references, is provided in Figures 3A,B.

##### 3.4.2.1 Mild and Moderate disease stages

These stages are initiated by SARS-CoV-2 infection, which triggers immune responses in respiratory epithelial cells and leads to the release of proinflammatory cytokines into the surrounding space. The damage caused by proinflammatory cytokines affects nearby microvascular endothelial cells, impairing capillary blood flow and causing low oxygen levels (hypoxia) in alveolocapillary units. Hypoxia activates hypoxia-inducible factors (HIFs), which shift the metabolism of hypoxic cells from OXPHOS to glycolysis. During this process, the upregulated LDH enzyme catalyzes the conversion of pyruvate to lactate, disrupting acid-base balance and lowering intracellular pH. At this early stage, when tissue pH remains above 6.7, lactate further promotes glycolysis by positive feedback on the HIF-1α transcription factor, which upregulates multiple glycolytic genes and enzymes.

##### 3.4.2.2 Deterioration

Deterioration occurs when a patient progresses from mild or moderate symptoms to severe respiratory distress and systemic inflammation. This shift occurs when accelerated SARS-CoV-2 replication triggers an uncontrolled inflammatory response, leading to hypercytokinemia and contributing to coagulopathy and microvascular thrombosis in the pulmonary circulation, worsening pulmonary hypoxia. When oxygen saturation falls below ca. 90%, the lung environment becomes hypoxemic. Hypoxemia then triggers a rapid increase in glycolysis that occurs throughout the lungs, causing a significant decrease in pH. In other systems, such as cancer, when pulmonary pH falls below 6.7, and acidosis becomes harmful, lactate’s feedback on HIF-1α turns negative, reducing glycolysis and increasing OXPHOS, thereby maintaining metabolic homeostasis (25, 26). However, during COVID-19 deterioration, this type of metabolic homeostasis is disrupted because the virus directly infects host cells, causing rapid and widespread mitochondrial dysfunction (mitochondriopathy). Since functional mitochondria are essential for regulating the entire OXPHOS machinery, mitochondriopathy disrupts metabolic homeostasis, prevents OXPHOS recovery, and leads to continuous lactate overproduction (lactic acidosis), which causes extensive tissue damage, pulmonary thrombosis, and cellular necrosis. Increased glycolysis results in more LDH being released into the bloodstream, while pulmonary thrombotic injury raises D-dimer levels (see Figures 3A,B, for more details).

##### 3.4.2.3 Severe and critical stages

Persistent hypoxemia and sustained inflammation progressively damage the lungs, eventually leading to ARDS, characterized by diffuse alveolar damage, low oxygen levels, stiff, noncompliant lungs, and bilateral infiltrates, which may develop into multiorgan failure (27). COVID-19–related ARDS results in death mainly due to unresolved lung injury, with in-hospital death rates often exceeding 30–50% in ICU patients. In COVID-related ARDS observational studies, pulmonary reasons were the most reported cause of death (up to 88%) (28).

## 4 Discussion

Our current work aims to elucidate the mechanisms of death in COVID-19, rather than merely identifying predictors of death. To achieve this, we focused on a subset of patients who advanced to a serious, life-threatening stage of the disease and compared those who deteriorated and then recovered with those who died. Understanding the cause of death in COVID-19 could help improve treatment for critically ill patients and provide insights into the complexities of other acute respiratory illnesses.

Our findings indicate that significant differences between survivors and nonsurvivors did not emerge prior to clinical deterioration. This contradicts previous reports suggesting that baseline blood values obtained at hospital admission can predict short-term mortality. This discrepancy may be due to earlier studies aligning patients by hospital admission date. Such an approach naturally compares blood variables among patients at different disease stages. In contrast, our method aligns patients by the point of deterioration, thereby comparing them at the same specific disease stage.

Our statistical analysis of the clinical results further indicates that LDH and D-dimer levels increased significantly during deterioration and during the first 2 weeks afterward in nonsurvivors. Among survivors, platelet counts increased markedly during the second week after deterioration. In the third week post-deterioration, the differences in these variables between survivors and nonsurvivors were insignificant, likely because most nonsurvivors had already died. All other clinical variables examined showed no distinction between the two patient groups at any stage of disease progression (Table 1). Our results indicate that the death of most patients with COVID-19, young and old alike, cannot be merely due to gradual tissue destruction by the virus. Instead, we suggest that death is determined by critical events taking place during patient deterioration. This result underscores the importance of focusing on the deterioration episode when analyzing the causes of death in COVID-19.

Supported by our results, we proposed a hypothetical model of post-deterioration death in patients (Figures 3A,B). Our model is based on four pillars: (a) as deterioration occurs, the gradually decreasing blood oxygen levels in the lungs cross a critical threshold, causing the lungs to become hypoxemic and triggering many healthy pulmonary cells to reprogram their metabolism to glycolysis simultaneously; (b) as a result, tissue glycolysis increases abruptly and significantly, boosting lactate production and secretion into the inter-alveolar space and exacerbating lactic acidosis; (c) shifting of metabolism back to OXPHOS, which would reduce lactic acidosis toxicity, is hindered by extensive virus-induced tissue mitochondriopathy; consequently, cells are trapped in a cycle of relentless glycolysis, high lactate levels, and widespread cell death; (d) lactic acidosis impairs tissue hemostasis, contributing to COVID-19 respiratory pathophysiology, ARDS, and death (29).

The dynamic changes in measured biochemical markers and blood counts among patients who are deteriorating support our proposed model. The biochemical markers CRP, LDH, and D-dimer are known to indicate systemic inflammation, tissue damage, and activation of the coagulation pathway, respectively. The protein CRP, synthesized in response to proinflammatory cytokines, is a nonspecific marker of systemic inflammation (30). We observe in Figure 2 that CRP blood levels rise during the week before deterioration, peak at the point of deterioration, and then decline, with no significant differences between survivors and nonsurvivors. This finding suggests a general similarity between the two groups in the inflammation process itself.

The blood levels of the glycolytic enzyme LDH are known to increase significantly when cells sustain damage from pathogenic insults, compromising cell membrane integrity and leading to extracellular LDH release (31). Our clinical results show a significant rise in LDH levels in the week following deterioration in nonsurvivors, which may reflect higher glycolytic rates, possibly leading to greater cellular damage in nonsurvivors (32), even under mechanical oxygenation. D-dimer is a degradation product of cross-linked fibrin produced when the fibrinolytic system breaks down fibrin clots. Elevated circulating D-dimer concentrations serve as markers of coagulation impairment, thrombotic events, and fibrin degradation (33), and Engström and colleagues show that lactic acidosis significantly impairs coagulation (34). We speculate that the markedly elevated D-dimer concentrations observed in nonsurvivors mirror an equally marked increase in lactic acidosis in these patients following an episode of massive glycolysis.

The blood count dynamics also support our hypothesis, showing a highly significant difference between survivors and nonsurvivors only in platelet counts. A temporary increase in platelet counts during deterioration, as seen in the nonsurvivors group (Figure 2A), may be caused by hypoxia-triggered “shedding” of platelets by megakaryocytes, as demonstrated in mice (35, 36). The decline in blood platelet counts in nonsurvivors after deterioration can be explained by significant platelet apoptosis and the aggregation of peripheral platelets into microthrombi, driven by elevated lactate levels at deterioration and the development of disseminated intravascular coagulation (37–39). Additionally, lactate is known to be an essential regulator of rapid neutrophil mobilization from the bone marrow into circulation during acute bacterial inflammation (40). A similar effect may be observed here, with an increase, albeit insignificant, in neutrophil counts in nonsurvivors during the 2 weeks following deterioration.

The current understanding is that multiple factors, including a weakened immune response, underlying health conditions, age, and the severity of organ damage, contribute to death in COVID-19. Based on our study, we hypothesize that a common underlying factor in the multifactorial causes of death in COVID-19 is the inability to maintain metabolic homeostasis in pulmonary tissue infected with SARS-CoV-2 during disease progression. As a result, lung cells become trapped in a harmful, endless glycolytic cycle. We suggest investigating whether impaired metabolic balance truly serves as the common denominator in these patient death scenarios.

The definitive understanding of COVID-19’s pathophysiology and questions about causality will likely await the development of animal models of the disease. However, at this stage, we can already analyze blood and solid tissue samples from survivors and nonsurvivors to examine differences in genomic, proteomic, or metabolomic expression related to glycolysis. In particular, the hypothesis that mitochondrial damage is a major driver of the irreversible transition to glycolysis should be tested in animal models. Confirming the effect of mutations on metabolic reprogramming could also lead to new COVID-19 treatments targeting those mutations.

Our study has certain limitations. The patient cohort was drawn from a single hospital repository over a relatively short period during the pandemic, which limits its diversity. However, since all patients received similar treatment upon clinical deterioration, treatment effects are not expected to account for differences between survivors and nonsurvivors. As for the conceptual model, high LDH levels in nonsurvivors cannot be attributed solely to excessive glycolysis, as LDH is released into the bloodstream whenever cell death occurs, regardless of the cause, and evidence indicates that other mechanisms, such as cytokine storm and inflammation, contribute to elevated LDH. Yet, cytokine storm intensity strongly correlated with CRP levels, which reflect ongoing inflammation (41). In our study, CRP increased with deterioration, but the difference between survivors and nonsurvivors was not statistically significant. Therefore, there is no reason to assume that LDH levels increased differentially in the nonsurvivors group exclusively due to their more intensive cytokine storm and greater inflammation. Other death causes in COVID-19 may involve myocardial damage, hepatic dysfunction, hemolysis, and renal impairment. These COVID-19 complications are also associated with dysregulation of glycolysis (9), supporting our hypothesis that, most plausibly, the elevated LDH levels in nonsurvivors are caused by dysregulated glycolysis. One might also suggest that variations in an unknown cell death mechanism could explain the differences in LDH levels among COVID-19 patients. However, the core of this putative mechanism, why it operates during clinical deterioration, and how it increases mortality, remains unknown. In contrast, our model is supported by studies across multiple tissues that consistently show a significant positive correlation between the LDH expression at both the mRNA and protein levels and the concentrations of the transcription factor governing glycolysis, HIF-1α. Additionally, evidence indicates that increased LDH activity can further stabilize HIF-1α, thereby reinforcing glycolysis and exacerbating lactic acidosis. Acidosis impacts cardiac contractility, vasomotor and enzyme activity, leading to hemodynamic instability and poorer outcomes (29, 42).

### 4.1 Future directions

Our hypothesis that excessive glycolysis during disease deterioration is a crucial fate-determining event in COVID-19 progression can be examined using antiglycolytic drugs, notably 2-DG, which is the only drug authorized (in India) as an emergency adjunct to standard care for moderate-to-severe COVID-19 (14). Antiglycolytic therapies like 2-DG are considered most suitable for hyperglycolytic states indicated by high LDH levels ( > 500 U/L) or other markers reflecting a metabolic shift from OXPHOS to glycolysis in COVID-19 pathology. However, the drug can have adverse effects on various systems of the body as it affects the normal metabolism of the cells because it targets cells non-selectively (14, 31). This therapy could suppress immune cell responses and lower the production of proinflammatory cytokines. Because of its modest clinical benefits, limited data, and safety concerns, glycolytic therapy has not been widely adopted (14).

Another such agent is the specific glucokinase activator AZD 1656, proposed for treating patients with diabetes and COVID-19 comorbidity due to its glucose-lowering effects and immunological mechanism of action. It has been noted that misuse of this and other antiglycolytic agents could risk immune suppression by starving glycolytic-dependent effectors, such as T cells and macrophages, leading to energy deficits, lymphopenia, or impaired interferon responses—especially during early or hypoxic phases, or in non-hyperglycolytic patients (43). Based on our findings, it may be worthwhile to investigate whether restricting the use of AZD 1656 or similar drugs to the critical phase of disease deterioration can maintain efficacy while reducing immunosuppressive effects. This proposition warrants further experimental investigation.

Previously, we developed a mathematical personalization algorithm that predicts imminent deterioration in patients with COVID-19 within 2–4 days before it occurs. We showed that it is possible to predict impending deterioration in nonsevere COVID-19 patients using a combination of eight routinely collected blood parameters, including neutrophil, lymphocyte, monocyte, and platelet counts; neutrophil-to-lymphocyte ratio; CRP, LDH, and D-dimer (44). We suggest using a deterioration-prediction algorithm, such as (44) to alert healthcare providers to the patient’s imminent deterioration and to time antiglycolytic therapy, possibly along with prophylactic mechanical ventilation. This could be an effective way to prevent metabolic collapse and reduce patient mortality. This approach should be thoroughly validated through proper experiments.

## 5 Conclusion

We hypothesized that a predominantly one-way metabolic shift, from OXPHOS to toxic glycolysis at the point of deterioration, combined with an inability to reverse this shift due to mitochondrial damage caused by the virus, creates a deadly trap for COVID-19 patients. Confirming the molecular mechanisms underlying this extreme metabolic imbalance is crucial for developing therapies that can effectively restore OXPHOS and improve treatment options. This could help identify key blood markers that distinguish potential survivors from nonsurvivors, thereby informing clinical decision-making and therapy planning for COVID-19 and other infectious diseases with similar features.

## Data Availability

The data analyzed in this study is subject to the following licenses/restrictions: No data repository is available for this study. Requests for the complete de-identified patient dataset addressed to the corresponding author will need to be reviewed by the Data Protection Officer of Sheba Medical Center. Requests to access these datasets should be directed to agur@imbm.org.
